# Recent progress in electrochemical decomposition of hydrogen sulfide for sulfur recovery and hydrogen production

**DOI:** 10.3389/fchem.2025.1698815

**Published:** 2025-11-14

**Authors:** Yanjun Chen, Ming Wen, Tong Ding, Rui Fan, Qisong Liu, Zongshe Liu, Zicheng Peng

**Affiliations:** 1 Research Institute of Natural Gas Technology, PetroChina Southwest Oil and Gasfield Company, Chengdu, China; 2 National R&D Center for High Sulfur Gas Exploitation, Chengdu, China; 3 High Sulfur Gas Exploitation Pilot Test Center, China National Petroleum Corporation, Chengdu, China; 4 PetroChina Southwest Oil and Gasfield Company, Chengdu, China

**Keywords:** electrochemistry, direct H_2_S decomposition, indirect H_2_S decomposition, sulfur recovery, hydrogen production

## Abstract

Hydrogen sulfide (H_2_S) generated by industrial processes (such as petroleum refining, natural gas purification, and coal processing) is a highly toxic and corrosive gas, which is detrimental to human health and environment. Electrocatalytic decomposition of H_2_S for simultaneous desulfurization and hydrogen production has emerged as a promising approach to addressing environmental pollution whilst achieving valuable utilization of H_2_S. Currently, there are two pathways for electrochemical decomposition of H_2_S, namely, direct and indirect decomposition. For the direct pathway, H_2_S is electrocatalytically oxidized into sulfur at anode using electrocatalysts. However, this approach is hindered by electrocatalyst deactivation due to sulfur passivation. Conversely, the indirect pathway effectively prevents the anodic sulfur passivation by introducing soluble redox couples as mediators, transferring H_2_S oxidation reaction from electrode to liquid phase. In this regard, the selection of redox mediators is critical since it affects H_2_S oxidation efficiency, sulfur purity, and overall decomposition voltage. In light of the challenges associated with above-mentioned electrochemical H_2_S decomposition techniques, this review presents recent advancements in strategies to mitigate anodic sulfur passivation for direct decomposition method, as well as the development of redox mediators and process optimization for indirect decomposition method. Meanwhile, a comparative analysis of characteristic and reaction mechanism of both approaches is provided. Finally, perspectives are given on the current challenges and future research directions in the field of electrocatalytic H_2_S splitting technology.

## 1 Introduction

H_2_S is a highly toxic and corrosive gas primarily generated from industrial processes such as petroleum refining, natural gas purification, and coal processing (De Crisci et al., 2019; Zheng et al., 2023). Waste gases containing H_2_S not only corrode oil and gas pipelines and equipment but also cause environmental pollution and seriously threaten human health when emitted into the atmosphere (Yu et al., 2024; Zhang et al., 2014). Consequently, the detoxification treatment of H_2_S gas is imperative. Currently, most refineries and natural gas purification plants employ the conventional Claus process to convert H_2_S into elemental sulfur and water through high-temperature combustion (925–1204 °C) followed by low-temperature catalytic reactions (170–350 °C) (Eow, 2002; Zhang et al., 2015; Zhao et al., 2021). However, this process generates a significant amount of sulfur-containing tail gas, necessitating additional tail gas treatment units and resulting in an extended process flow (Eow, 2002; Zhao et al., 2021). Moreover, while the Claus process recovers sulfur, it oxidizes valuable hydrogen resources into water, leading to a substantial waste of hydrogen energy. In the context of carbon neutrality and the development of green energy, various strategies have been explored for the simultaneous recovery of sulfur and hydrogen from H_2_S, encompassing thermal decomposition, electrocatalytic decomposition, plasma-assisted decomposition, photocatalytic decomposition, and microwave-induced decomposition approaches (Basheer and Ali, 2019; Chen et al., 2020; Khabazipour and Anbia, 2019; Li J. et al., 2021; Oladipo et al., 2021; Osasuyi et al., 2022; Sassi and Amira, 2012; Wang S. et al., 2024; Zhang et al., 2024).

Electrochemical decomposition of H_2_S demonstrates superior advantages over alternative technologies, featuring mild reaction conditions (room temperature and atmospheric pressure), low energy requirements, high efficiency, simple operation, and spatially separable hydrogen/sulfur products, representing a promising route for clean and value-added H_2_S utilization (Yang et al., 2023; Zhou et al., 2018). By constructing a multi-stage electrolysis system, this technology not only enables effective removal of H_2_S within a wide concentration range, but also produces green hydrogen and elemental sulfur simultaneously, displaying a one-stone-kills-two-birds strategy for environmentally friendly and economic benefits (Pei et al., 2021). Electrocatalytic splitting of H_2_S technology comprises two approaches, namely, direct and indirect H_2_S decomposition. The direct electrocatalytic decomposition of H_2_S occurs at the anode electrode, with a theoretical decomposition voltage (∼0.17 V vs. SHE) that is significantly lower than that of water electrolysis (1.23 V) (Kim and Lee, 2023; Zhang et al., 2022). Nevertheless, it faces challenges, including low activity of catalytic materials and sulfur passivation in the sulfur oxidation reaction on electrodes (Yi et al., 2021). In contrast, the indirect decomposition of H_2_S employs redox couples as mediators. In this process, sulfur ions are oxidized by redox mediator to elemental sulfur (Zhou et al., 2018). This approach circumvents anode sulfur passivation but encounters issues such as elevated cell voltage and mismatched reaction rates between sulfur oxidation and electrochemical processes; thus, the selection of redox mediators is essential.

**FIGURE 1:**
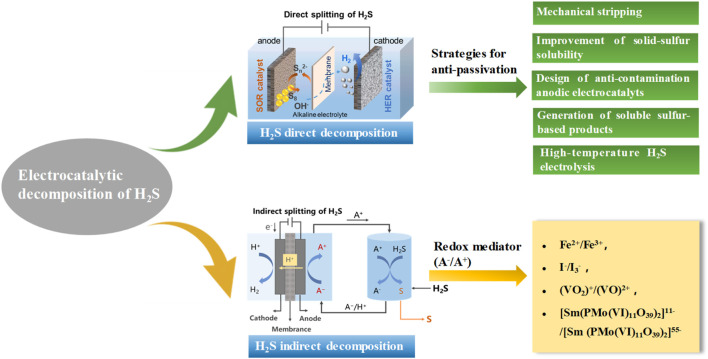
Schematic diagram of methods and strategies for electrocatalytic H_2_S decomposition.

Currently, both methods for H_2_S decomposition have attracted broad research attention and yielded promising results. In response to the challenges associated with the aforementioned two electrochemical H_2_S decomposition technologies, this review summarizes recent research progress on anti-sulfur passivation strategies for direct decomposition method, as well as advancements in redox mediator selection and process optimization for indirect decomposition method. Meanwhile, a detailed comparison is presented between the two methods in terms of their key characteristics and reaction mechanisms (Figure 1). Lastly, the current challenges and future research directions in the field of electrocatalytic H_2_S splitting technology are explored, with the expectation of providing a reference for the research and development of H_2_S treatment and resource utilization technologies generated in the purification process of sulfur-containing natural gas.

## 2 Direct electrochemical decomposition of H_2_S

### 2.1 Mechanism

**FIGURE 2:**
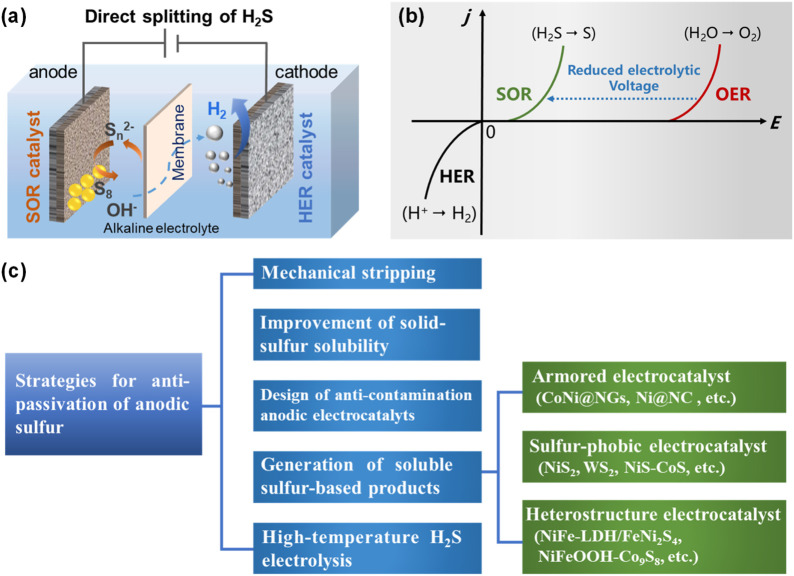
**(a)** Schematic illustration of directly H_2_S electrolysis, **(b)** the electrochemical potentials of SOR for H_2_S splitting over OER for typical water splitting, and **(c)** strategies for anti-passivation of anodic sulfur (Garg et al., 2023; Yu et al., 2024).

The direct electrochemical H_2_S splitting involved the sulfide oxidation reaction (SOR) to produce elemental sulfur (Equations 1, 2) at anode and the hydrogen evolution reaction (HER) to produce hydrogen (Equation 3) at cathode (Figure 2a) (Garg et al., 2023). Usually, an alkaline electrolyte was used to dissolve H_2_S.

$$\text{Anode}:{\text{HS}}^{‐}+{\text{OH}}^{–}\;\to \;S+H_{2}O+2e^{–}$$ (1)

$$S^{2‐}\;\to \;S+2e^{‐}$$ (2)

$$\text{Cathode}:2H_{2}O+2e^{‐}\;\to \;H_{2}+{\text{OH}}^{‐}$$ (3)

The theoretical voltage of directly electrocatalytic splitting of H_2_S is lower than oxygen evolution reaction (OER, 1.23 V) in alkaline electrolyte, which is an effective method for the treatment and utilization of H_2_S (Figure 2b). However, the deposition of sulfur on the anode during the process creates a passivation layer with extremely high resistivity (∼10^15^ Ω m), which physically obstructs access to the catalytically active sites by sulfide species, resulting in progressive active site deactivation and complete reaction inhibition. Meanwhile, due to the possible existence of various sulfur-containing oxidation species (polysulfides, 0/−1; element sulfur, 0; sulfite, +4; thiosulfate, −1/+5; sulfate, +6), the sulfur oxidation reaction process in aqueous electrolytes was complex, involving multiple sulfide-containing intermediates and branched-chain reactions (Equations 4–9), which resulted in the complexity of anode reaction products (Anani et al., 1990; Ateya et al., 2003; Kim and Lee, 2023).

$${\text{HS}}^{‐}+\left(n‐1\right)\;S+{\text{OH}}^{–}\;\to \;S_{n}^{2‐}+H_{2}O;\;n=2∼5$$ (4)

$$S+S_{n}^{2‐}\;\to \;S_{n+1}^{2‐};\;n=2∼4$$ (5)

$$2{\text{HS}}^{‐}+8{\text{OH}}^{‐}\;\to \;S_{2}O_{3}^{2‐}+5H_{2}O+8e^{‐}$$ (6)

$$S^{2‐}+6{\text{OH}}^{‐}\;\to \;{{SO}_{3}}^{2-}+3H_{2}O+6e^{‐}$$ (7)

$$S_{n}^{2‐}+6{\text{OH}}^{‐}\;\to \;S_{2}O_{3}^{2‐}+3H_{2}O+\left(n‐2\right)\;S+6e^{‐};\;n=2∼5$$ (8)

$$S+4H_{2}O\;\to \;{\text{SO}}_{4}^{2‐}+8H^{+}+6e^{‐}$$ (9)

To address the issue of anode sulfur passivation, early-stage research primarily focused on employing methods (Figure 2c) such as mechanical stripping and improvement of solid-sulfur solubility to rapidly remove reaction-generated elemental sulfur from the electrode surface, thereby preventing sulfur-induced electrode passivation (Ma et al., 2020; Shih Y S, 1986). In recent years, with the development of catalytic materials and in-depth understanding of the SOR process, researchers have proposed to solve the sulfur passivation problem by designing anti-contamination anodic electrocatalyts for SOR (such as armored electrocatalytic materials, sulfur-phobic electrocatalytic materials, etc.) and generation of soluble sulfur-based products (Wei and Wu, 2025; Zhang B. et al., 2021; Zhang et al., 2025). In addition, high-temperature H_2_S electrolysis with sulfur being recoverable in gaseous form to prevent solid sulfur deposition at anode has also been developed (Ipsakis et al., 2015).

### 2.2 Strategies for anti-passivation of anodic sulfur

#### 2.2.1 Mechanical stripping

Mechanical stripping is a method that physically removes the adherents on the surface of the catalyst through external force. The early study demonstrated that the use of mechanical stripping to remove the adhered sulfur at the anode for SOR is feasible. Shih et al. achieved continuous removal of the generated sulfur from the anode by designing the CSTER system (Shih, 1986), a continuously stirred electrochemical reactor, with organic solvent as sulfur dissolver, in which the organic solvent (e.g., toluene or benzene) is mixed with the alkaline sulfide solution by means of continuous stirring to achieve sulfur recovery efficiencies of up to 80% from the anode at ambient temperature and pressure.

#### 2.2.2 Improvement of solid-sulfur solubility

**FIGURE 3:**
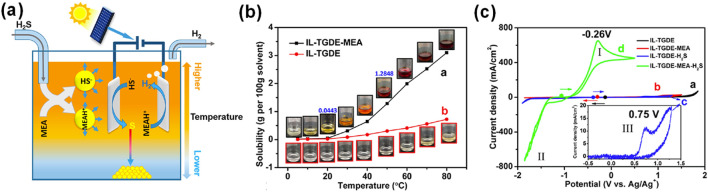
Improvement of solid-sulfur solubility. **(a)** Conceptual schematic of H_2_S continuous electrolysis with organic electrolyte system, **(b)** solubility of sulfur in organic systems for IL−TGDE−MEA and IL−TGDE at different temperatures, and **(c)** CV curves of a Pt microdisk electrode with a diameter of 30 μm at 50 °C and a scan rate of 100 mV/s at four organic electrolyte system (Ma et al., 2020).

Organic solvents such as ionic liquids used as electrolytes have been demonstrated to have significant potential in enhancing sulfur solubility while maintaining favorable electrical conductivity (Li et al., 2024; Ma et al., 2017; Wang et al., 2019; Yang et al., 2025). Ma et al. constructed an organic electrolyte system for the direct electrolysis of H_2_S using ionic liquid [C_3_OHmim]BF_4_ as the electrolyte, tetraethylene glycol dimethyl ether (TGDE) as the solvent, and monoethanolamine (MEA) as the absorbent for H_2_S (Figure 3a). The research finds that the prepared organic electrolyte system is highly temperature-dependent on the sulfur solubility electrolyte and has the catalytic effect of the absorbent MEA. As shown in Figure 3b, due to the solubility of sulfur in organic solutions varying greatly with temperature, the high temperature zone near the anode can maximize the dissolution of precipitated sulfur in the reaction process, and the subsequent low temperature zone away from the electrode makes the sulfur precipitation, thus recovering sulfur. Meanwhile, the reaction of MEA with H_2_S would produce protonated MEA (MEAH^+^) and HS^−^ (MEA + H_2_S → MEAH^+^ + HS^−^), which not only increases the solubility of H_2_S in the electrolyte system, but also makes it easier for H_2_S to be electrolyzed into HS^−^ and promotes the oxidation of HS^−^. As a result, the addition of MEA led to a decrease in the oxidation potential of H_2_S from 0.75 V to −0.26 V, improving the electrolysis efficiency (Figure 3c). Highly efficient and continuous electrolysis of H_2_S can be achieved in this organic electrolyte system with Faraday efficiency of H_2_ up to 89%.

#### 2.2.3 Design of anti-contamination anodic electrocatalysts

Designing SOR electrocatalysts with high catalytic activity, anti-passivation of sulfur (referring to the ability of the catalyst to prevent sulfur from adhering and causing catalyst deactivation), and long-term stability is an effective strategy to mitigate anodic sulfur passivation in electrocatalytic decomposition of H_2_S (Kim and Lee, 2023). Recently, researchers have developed SOR electrocatalysts mainly from the following aspects (Yu et al., 2024): (1) Constructing a sulfur-phobic interface by controlling the catalyst’s affinity for sulfur to prevent the product sulfur from adhering to the catalyst surface, facilitating the self-cleaning effect of the electrocatalyst. (2) Modulating the electronic structure of the SOR catalysts by surface modification, alloying, heterojunction construction, and core-shell architecture, could optimize the adsorption and desorption free energies of sulfur-containing intermediates, ensuring the optimum binding between the active catalyst and reaction intermediates, thus improving the catalytic activity while preventing anodic sulfur passivation.

**FIGURE 4:**
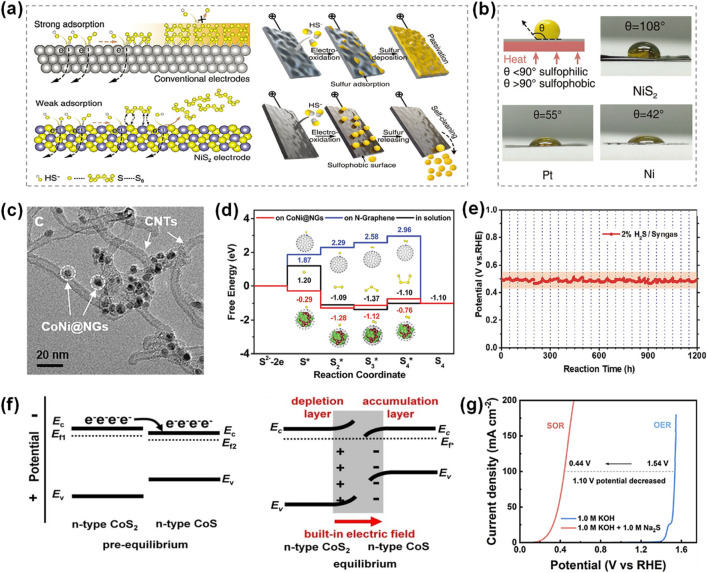
Design of anti-contamination anodic electrocatalyst. **(a)** Strong interaction between electrodes and sulfides leads to sulfur passivation during long-term operation, whereas weak interactions repel sulfur and realize self-cleaning electrolysis, and **(b)** the contact angle measurements of sulfur droplets (120 °C) on NiS_2_, Pt and Ni (Zhang B. et al., 2021). **(c)** HRTEM images of CoNi@NGs armored catalyst, **(d)** the free energy profiles of the formation of polysulfides (S_x_*) in the aqueous solution on N-Graphene’s surface and CoNi@NGs’ surface, and **(e)** durability measurement of CoNi@NGs for removing H_2_S in industrial syngas via flowing with 2% H_2_S/syngas (Zhang et al., 2020). **(f)** Schematic diagrams of the energy band structures of the over-equilibrium and equilibrium states of the CoS_2_/CoS n-n heterojunction, and **(g)** the LSV curves of NiFe-LDH/FeNi_2_S_4_/IF (Ma W. et al., 2019).

Currently, SOR electrocatalysts include metal oxides, metal sulfides, transition metal selenides/tellurides, metal alloy catalysts, etc. has been reported (Ali et al., 2022; Yu et al., 2024). Table 1 exhibits electrocatalytic performance for recently reported SOR catalysts. Zhang et al. discovered the sulfur-phobic property of transition metal sulfides surface towards sulfur species and developed self-cleaning NiS_2_ electrocatalysts through a vapor-phase sulfurization method, leveraging sulfur-phobic interface design and controlled sulfur vacancy incorporation (Zhang S. et al., 2021). This NiS_2_ catalyst allows for efficient SOR (1.05 g_sulfur_ W h^-1^) and simultaneous hydrogen production (0.07 g W h^-1^ H_2_) with low energy consumption. Theoretical calculations and experimental results demonstrate that NiS_2_ is susceptible to the adsorption/desorption of sulfur, ensuring that the generated sulfur will not adhere to the electrode surface (Figures 4a,b). The assembled electrolytic system of H_2_S requires an ultralow cell voltage of only 0.65 V to provide a current density of 20 mA cm^-2^ and stable operation for more than 100 h. Additionally, similar sulfur-phobic self-cleaning materials such as WS_2_ nanosheets prepared by a molten salt-assisted method (Yi et al., 2021) and NiS-CoS nanosheet composites synthesized by a hydrothermal method (Huo et al., 2023) have also been reported.

**TABLE 1:** Comparison of SOR performance for recently reported catalysts.

Catalyst	Electrolyte	Potential @ 100 mA cm^-2^	Stability test	Reference
Fe, F-NiO	1 M KOH + 1 M Na_2_S	0.63 V	100 h@ 1.65 V	*Small*, 2023, 19, 2302055
Cu_2_S/NF	1 M Na_2_S + 1 M NaOH	0.44 V	48 h@ 50 mA cm^-2^	*Green Chem.*, 2021, 23, 6975
Co_3_O_4_	1 M Na_2_S + 1 M NaOH	0.26 V	72 h@ 100 mA cm^-2^	*Adv. Funct. Mater.*, 2022, 33, 2212183
n-Co_3_S_4_@NF	1 M Na_2_S + 1 M NaOH	0.233V	240 h@ 0.524 V	*Nat. Commun.*, 2024, 15, 6173
Co-Ni_3_S_2_	1 M Na_2_S + 1 M NaOH	0.59 V	24 h@ 50 mA cm^-2^	*Chem. Eng. J.*, 2022, 433, 134472
NiCu-MoS_2_	1 M NaOH	0.35 V	30 h@ 0.3 V	*J. Mater. Chem. A*, 2022, 10, 13031
Mo-Co-S/NF	1 M Na_2_S + 1 M NaOH	0.29 V	40 h@ 0.25 V	*Inorg. Chem. Front.*, 2023, 10, 6728
Ni-Co-C/NF	1 M Na_2_S + 1 M NaOH	0.336 V	64 h@ 100 mA cm^-2^	*Inorg. Chem. Front.*, 2023, 10, 1447
FeMo-S/Ru	2.43M Na_2_S +1M NaOH	0.3 V	45 h@ 10 mA cm^-2^	*Adv. Funct. Mater.*, 2024, 34, 2315326
Co_3_O_4_@WS_2_	4 M Na_2_S + 1 M NaOH	0.6 V	24 h at 10 mA cm^-2^	*J. Mater. Chem. A*, 2024, 12, 29184
V_Pd_-Pd_4_S MNRs	4 M Na_2_S + 1 M KOH	0.65 V	20 h@ 10 mA cm^-2^	*Appl. Catal. B Environ.*, 2024, 340
CoNi@NGs	1 M Na_2_S + 1 M NaOH	0.52 V	500 h@ 0.317 V	*Energy & Environ. Sci.*, 2020, 13, 119
CuCoNiMnCrS_x_	3 M Na_2_S + 1 M NaOH	0.25 V	120 h@ 1000 mA cm^-2^	*Angew. Chem. Int. Ed.,*2024, e202411977
HEA-Mo_2_C/HPC	1 M Na_2_S + 1 M NaOH	0.38 V	210 h@ 1000 mA cm^-2^	*ACS Nano*, 2023, 17, 25707
NiSe/NF	1 M Na_2_S + 1 M NaOH	0.49 V	500 h@ 0.4 V	*Appl. Catal. B Environ.*, 2023, 324, 122255
MoSe_2_/Ni_x_Se_y_	1 M Na_2_S + 1 M NaOH	0.5 V	48 h@ 100 mA cm^-2^	*Fuel*, 2024, 374, 132532
Ru-CoSe	1 M Na_2_S + 1 M NaOH	0.273 V	100 h@ 120 mA cm^-2^	*Small*, 2024, 20, 2,406,012
NiMoN/MNF	1 M Na_2_S + 1 M NaOH	0.33 V	100 h@ 300 mA cm^-2^	*Adv. Funct. Mater.*, 2024, 34, 2407601
NiFe-LDH /Cu-IF	1 M Na_2_S + 1 M NaOH	0.3 V	120 h@ 50 mA cm^-2^	*Fuel*, 2024, 367, 131506

Furthermore, Researchers proposed an armored catalyst strateges such as graphene-encapsulated metal to prevent sulfur passivation (Yu et al., 2022). This approach involves encapsulating transition metal with two-dimensional layered materials, creating well-defined core-shell nanostructures. The two-dimensional layer serves as a protective barrier that effectively prevents electrode corrosion and surface passivation, thereby maintaining catalytic activity. Zhang et al. fabricated a nitrogen-doped graphene-encapsulated non-precious CoNi nanoalloy (CoNi@NGs) by a template-assisted method as the effective electrocatalyst for simultaneous hydrogen and sulfur production from H_2_S (Figure 4c) (Zhang et al., 2020). The CoNi@NGs armored catalyst exhibits excellent activity with a potential of 0.25 V for driving SOR, which is 1.24 V lower than that for OER, suggesting that the energy required to decompose H_2_S is much lower than that required to decompose water. Theoretical calculations revealed that the synergy between the encapsulated metal alloy and doped nitrogen modulated the electronic structure of graphene shells, promoting the adsorption of sulfur-containing intermediates (S*) and the formation of polysulfide intermediates on the graphene surface, thus preventing sulfur passivation and inducing high SOR activity (Figure 4d). Meanwhile, the prepared CoNi@NG catalyst was used for the direct electrolysis of H_2_S in industrial syngas (49% CO, 49% H_2_, and 2% H_2_S), which displayed excellent activity and high stability for more than 1200 h at a current density of 20 mA cm^-2^ (Figure 4e). Moreover, Zhang et al. reported an integrated electrode with dual-level chainmail structure to facilitate the decomposition of H_2_S (Zhang et al., 2025). The primary chainmail is created by a graphene-coated nickel foam skeleton, and the secondary chainmail is formed by graphene-encapsulated nickel nanoparticles. This integrated-chainmail electrode (Ni@NC foam) exhibits an excellent activity and stability for H_2_S splitting, which supplies an industrial-scale current density of 1 A cm^-2^ at 1.12 V, and can operate stably for more than 300 h at 100 mA cm^-2^. Additionally, the Ni@NC foam catalyst realizes the complete removal of 20% H_2_S in the simulated crude natural gas (80% CH_4_ and 20% H_2_S) at the anode to obtain sulfur, and simultaneous acquisition of high-purity hydrogen at the cathode.

The construction of heterostructure is another effective strategy for designing SOR electrocatalysts. The electron redistribution would occur at heterogeneous interfaces due to the Fermi level equilibration between constituent materials, thus modulating the local electronic environment and optimizing the adsorption free energy of sulfur-containing intermediates, thus enhancing the electrocatalytic performance. (Figure 4f) (Ma D. et al., 2019). Ai et al. successfully fabricated heterostructure nanoarrays of NiFe layered double hydroxide (LDH)/FeNi_2_S_4_ grown on iron foam (IF) substrate (Ai et al., 2024). The obtained NiFe-LDH/FeNi_2_S_4_/IF catalyst can effectively catalyze SOR with a low potential of only 0.44 V at 100 mA cm^-2^, due to the good hydrophilic surface and the good heterojunction interface (Figure 4g). Semwal et al. developed NiFeOOH-Co_9_S_8_ intercalated nanostructure arrays with varying Fe: Co ratios grown on nickel foam (NF) substrate by one-step method at low-temperature (50 °C) (Semwal et al., 2023). The NiFeOOH-Co_9_S_8_ heterojunction catalyst required only 0.84 V to achieve an industrial-grade current density of 1 A cm^-2^ in the coupled SOR-HER system. Jin et al. reported the synthesis of terephthalic acid (TPA)-anchored Ni_3_S_2_ (TPA-Ni_3_S_2_) brush-like nanoarrays on NF substrate (Jin et al., 2022). The introduction of TPA ligands facilitated the formation of a unique nanorod @nanosheet interfacial structure, which not only modulated the electronic structure of Ni_3_S_2_ to optimize the adsorption strength of sulfur-containing intermediates but also increased the number of active sites, thereby enhancing the catalytic performance for SOR.

#### 2.2.4 Generation of soluble sulfur-based products

**FIGURE 5:**
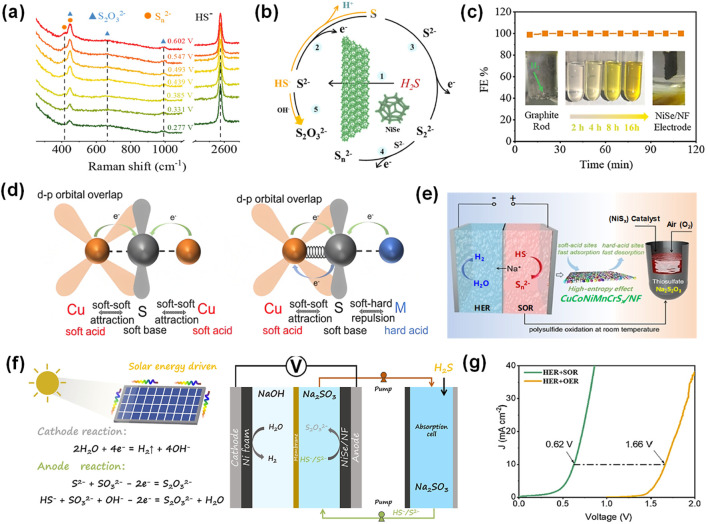
Strategies for the generation of soluble sulfur-based products. **(a)** *In situ* Raman spectra in the range of 250–2800 cm^-1^ at a potential between 0.277 and 0.602 V on NiSe/NF electrode in 1.0 M Na_2_S and NaOH solution, **(b)** The schematic diagram of the SOR reaction pathway on NiSe/NF, **(c)** Hydrogen faradaic efficiencies in galvanostatic test at 100 mA cm^-2^ (Duan et al., 2023). **(d)** Schematic illustration of the Cu-S bonds with/without doping of the hard-acid cations (denoted as M), **(e)** Schematic illustration of direct H_2_S decomposition for yielding Na_2_S_2_O_3_ products (Pei et al., 2024). **(f)** Direct solar-driven electrochemical decomposition of H_2_S to hydrogen and high-value sulfur product, **(g)** LSV curves of the hybrid HER and SOR of NiSe/NF (Duan et al., 2024).

Recent studies demonstrate that the products distribution of anodic SOR in electrocatalytic decomposition of H_2_S can be precisely controlled by optimizing key operational parameters, including applied potential window and electrolyte pH, in conjunction with rational electrode material design. Soluble sulfur-based products for SOR can be obtained through the tailored reaction pathway, effectively alleviating anodic sulfur passivation. The distribution of sulfur-based products at anode is intrinsically linked to the reaction mechanism of SOR. However, the mechanistic understanding of the SOR during the electrocatalytic decomposition of H_2_S remains incomplete. To address the complexity of SOR mechanisms on electrode materials, Duan et al. successfully constructed NiSe_2_/NF nanoarrays on nickel foam (NF) via a hydrothermal method for electrocatalytic H_2_S decomposition (Duan et al., 2023). By employing *in situ* characterization techniques, including Fourier-transform infrared spectroscopy and Raman spectroscopy, they elucidated the sulfur ion oxidation pathway (Figure 5a). When employing 1 mol/L NaOH-Na_2_S solution as the H_2_S absorbent, the selective conversion of HS^−^/S^2-^ to polysulfides (S_n_^2-^, n > 2) and the byproduct S_2_O_3_^2-^ rather than elemental sulfur was achieved (Figures 5b,c), effectively preventing electrode sulfur passivation during prolonged operation. Subsequently, the electrochemically generated polysulfides were converted to elemental sulfur through acidification in an ice bath using concentrated sulfuric acid. The experimental results show that the NiSe_2_/NF catalyst can operate stably for 500 h at 0.49 V. Additionally, Yang et al. systematically investigated Ni as the anode material for SOR (Yang et al., 2019). Their studies revealed that significant sulfur passivation occurred at the anode, during H_2_S electrolysis in Na_2_HPO_4_ solution (pH 9.2), In contrast, when operated in strongly alkaline media (pH 13), the formation of passivating sulfur layers was effectively suppressed. These results demonstrate that a higher pH condition preferentially inhibits sulfur passivation.

Furthermore, Pei et al. developed an amorphous high-entropy sulfide catalyst of CuCoNiMnCrS_x_ nanosheets for SOR, which provided a current density of 100 mA cm^-2^ at an ultra-low potential of 0.25 V, and maintained stable operation for 100 h at an industrial-grade current density of 1 A cm^-2^ (Pei et al., 2024). The superior SOR performance is attributed to the tailored chemical environment around Cu^+^ sites by soft/hard metal cations and the designed “soft acid” (Cu^+^) to “hard acid” (Co^2+^/Ni^2+^, Mn^2+^/Cr^3+^) active sites, which synergistically promote sulfur adsorption/desorption and facilitate the efficient conversion of S^2-^ into S_n_^2-^, preventing sulfur passivation at anode (Figure 5d). Particularly, this work designed an electrochemical-chemical tandem oxidation strategy to further convert S_n_^2-^ into higher-value thiosulfate over NiS_x_ catalyst (Figure 5e). Subsequent concentration, filtration, and crystallization yield high-purity sodium thiosulfate pentahydrate crystals (Na_2_S_2_O_3_·5H_2_O). This approach effectively circumvents the economic limitation of conventional acidification-based sulfur precipitation recovery.

Additionally, Duan et al. proposed a strategy for directional control of SOR products through adding reaction medium (Duan et al., 2024). The S^2-^/HS^−^ can be oxidized into high-valued Na_2_S_2_O_3_ via a one-step method using NiSe as catalyst in Na_2_SO_3_ media solution (Figure 5f), which effectively avoids sulfur passivation and complex sulfur recovery, achieving a current density of 200 mA cm^-2^ at a lower potential of 0.66 V (Figure 5g). The reaction pathway was verified by *in situ* Fourier-transform infrared spectroscopy and Raman spectroscopy, which provided real-time monitoring evidence for the following surface electrochemical reactions (Equations 10, 11):

$$2{\text{HS}}^{‐}+{\text{SO}}_{3}^{2‐}\;\to \;S_{2}O_{3}^{2‐}+H^{+}+2e^{‐}$$ (10)

$$S^{2‐}+{\text{SO}}_{3}^{2‐}\;\to \;S_{2}O_{3}^{2-}+2e^{‐}$$ (11)

#### 2.2.5 High-temperature H_2_S electrolysis

**FIGURE 6:**
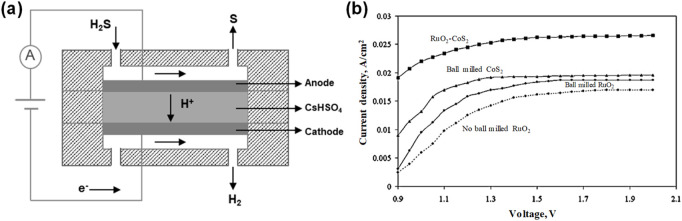
High-temperature H_2_S electrolysis. **(a)** Schematic of high-temperature solid electrolyte cells for H_2_S decomposition, and **(b)** current density for different anode configurations with 100% H_2_S feed gas content (Mbah et al., 2010).

Except for aqueous electrolytes, high-temperature solid electrolyte cells for the decomposition of H_2_S have also been initially developed. H_2_S-containing gas is oxidized at the anode of a high-temperature solid molten electrolyte cell to obtain sulfur and protons, in which sulfur is discharged in the form of sulfur vapor from the anode chamber at high temperature, and then cooled and concentrated to collect sulfur at the outlet, thus preventing sulfur from being enriched at the anode electrode and prompting continuous anodic reaction (Ipsakis et al., 2015). Meanwhile, protons go through the solid electrolyte membrane to reach the cathode chamber, being reduced to hydrogen. The reported proton-conducting solid electrolytes include LiSO_4_, CsHSO_4_, (ZrO_2_)_0.92_(Y_2_O_3_)_0.08_, Y-CeO_3_, BaCeO_3_, etc. (Yates and Winnick, 1999; Chuang, et al., 2000; M. Liu et al., 2001; Sezer et al., 2020). Mbah et al. synthesized a RuO_2_-CoS_2_ nanocomposite as an efficient anode catalyst for electrochemical H_2_S decomposition (Mbah et al., 2010). They assembled an H_2_S decomposition cell employing CsHSO_4_ as a high-temperature molten solid-state electrolyte (Figure 6a). Remarkably, under optimized conditions of 150 °C and 1.35 bar where sulfur viscosity was minimized, the system could achieve the electrolytic splitting of H_2_S (100% content) (Figure 6b). The high-temperature solid electrolyte cells could exhibit exceptional H_2_S decomposition efficiency, due to the high ionic conductivity of the molten electrolyte, accelerating the electrochemical reaction kinetics. Furthermore, this approach facilitates the efficient separation of the obtained hydrogen and elemental sulfur, with sulfur being recoverable in gaseous or liquid form, thus preventing electrode blockage caused by solid sulfur deposition.

Although strategies such as modulating reaction process parameters, selecting appropriate organic solvents as electrolytes, and designing SOR catalytic materials have partially addressed the sulfur passivation of electrode in the direct electrocatalytic decomposition of H_2_S, challenges such as the susceptibility of electrode materials to sulfidation at high current density, poor material stability, and low sulfur recovery efficiency still persist. Furthermore, due to the complex forms of sulfur in different electrolytes and the diverse existence of SOR products, the obtained sulfur species are complex. The targeted modulation to obtain a single sulfur-based chemical product is crucial for the valorization of sulfur resources and represents a pivotal step toward the industrial application of this technology.

## 3 Indirect electrochemical decomposition of H_2_S

### 3.1 Mechanism

**FIGURE 7:**
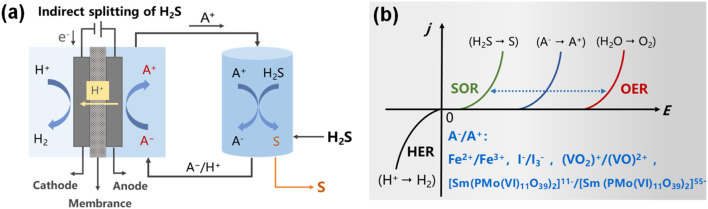
**(a)** Schematic illustration of indirect H_2_S electrolysis (Zhou et al., 2018) and **(b)** the electrochemical potentials of redox mediators for H_2_S splitting (Kim and Lee, 2023).

The indirect electrocatalytic facilitation of the H_2_S splitting into hydrogen and elemental sulfur was achieved by means of redox mediators in two consecutive steps: absorption oxidation and electrocatalytic regeneration (Figure 7a) (Zhou et al., 2018). In the absorption reactor (Equation 12), the oxidized-state mediator (A^+^) in the liquid absorbent solution oxidizes H_2_S into elemental sulfur, while being reduced to its reduced-state mediator (A^−^). During the electrochemical regeneration, the reduced-state mediator is converted into a reusable oxidized form at anode, while hydrogen products are obtained at the cathode (Equations 13, 14). Compared to the direct electrocatalytic decomposition of H_2_S, the anodic sulfur passivation can be fundamentally prevented in redox-mediated H_2_S splitting, due to the sulfur precipitation occurring at absorption reactor rather than on the electrode, simultaneously achieving the recycling of redox mediator.

$$\text{Oxidation of }H_{2}S:A^{+}+H_{2}S\;\to \;A^{‐}+2H^{+}+S↓$$ (12)

$$\text{Oxidation at anode}:A^{‐}\;‐\;2e^{‐}\;\to \;A^{+}$$ (13)

$$\text{Reduction at cathode}:2H^{+}+2e^{‐}\;\to \;H_{2}↑$$ (14)

where A^−^ and A^+^ are the reduced and oxidized states of the redox mediator, respectively.

In the indirect electrocatalytic decomposition of H_2_S system, the selection of a redox mediator is of paramount importance as it governs two key process parameters: H_2_S conversion efficiency and elemental sulfur purity. Generally, the selection of a redox mediator should satisfy the following criteria: (1) The redox medium must possess an appropriate redox potential (as shown in Figure 7b), which should be sufficiently high higher than that of H_2_S oxidation but lower than that of the OER, to oxidize H_2_S to elemental sulfur while avoiding excessive oxidation to generate by-products (SO_3_^2-^, S_2_O_3_^2-^ et al.). (2) The produced sulfur should be easily separable. (3) Both the oxidized and reduced states of the redox medium should be stable throughout the reaction and exhibit non-toxicity as well as environmental friendliness. Recently, many redox mediators such as (VO_2_)^+^/(VO)^2+^, I^−^/I_3_^−^, Fe^2+^/Fe^3+^ and polyoxometalates [Sm(PMo^IV^O_39_)_2_]^11-^/[Sm(PMo^V^O_39_)_2_]^55-^ have been reported (Huang et al., 2009; Kim et al., 2014; Zhou et al., 2018; Zong et al., 2014).

### 3.2 Redox mediators for indirectly electrocatalytic decomposition of H_2_S

#### 3.2.1 Fe^2+^/Fe^3+^ as redox mediators

**FIGURE 8:**
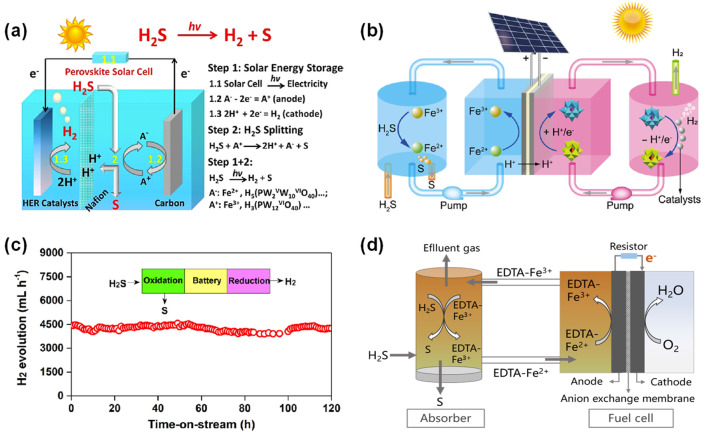
Fe^2+^/Fe^3+^ as redox mediators for indirect electrocatalytic decomposition of H_2_S. **(a)** Operating principle of the perovskite PV-EC H_2_S splitting system (Ma et al., 2016). **(b)** Schematic illustration of the solar redox flow batteries design for indirectly driving H_2_S splitting (Ma W. et al., 2019). **(c)** Stability test for H_2_S splitting into hydrogen and sulfur with the off-field electrocatalysis system (Wang S. et al., 2024). **(d)** A schematic representation of the proposed liquid redox sulfur recovery process with EDTA ligands and fuel cell (Kim et al., 2013).

The Fe^2+^/Fe^3+^ (0.77 V vs. SHE) was the earliest employed as redox mediator for indirect electrocatalytic decomposition of H_2_S to produce sulfur and hydrogen, owing to its cost-effectiveness, strong oxidative capability toward H_2_S, and facile electrochemical regeneration (Mizuta et al., 1991; Zhou et al., 2018). In 1991, Mizuta et al. first employed ferric chloride (FeCl_3_) solution to oxidize H_2_S, generating elemental sulfur, ferrous chloride (FeCl_2_), and hydrochloric acid (HCl) (Mizuta et al., 1991). Subsequently, the electrolyte solution with FeCl_2_ and HCl after separating sulfur precipitate was introduced to the electrolysis system, where Fe^2+^ was oxidized to Fe^3+^ at the anode, while H^+^ was reduced at the cathode to produce hydrogen. Experimental results demonstrated that under conditions of 70 °C and an electrolysis voltage of 0.7 V, the absorption efficiency of H_2_S exceeded 99%. Huang et al. assembled a bipolar membrane electrolysis device using graphite cloth as the anode and platinum-coated graphite cloth as the cathode, with a proton exchange membrane for the electrochemical regeneration of Fe^2+^/Fe^3+^ in an indirect H_2_S decomposition system (Huang et al., 2019). This bipolar electrolysis device exhibited a compact structure and high electrolytic efficiency. Experimental results demonstrated that the current density varied with electrolytic voltage, temperature, and electrolyte composition. When [Fe^3+^] > 0.20 mol/L, the concentrations of Fe^2+^ and Fe^3+^ in the anolyte showed no significant influence on the current density. Furthermore, Li’s group constructed a perovskite photovoltaic-electrocatalytic (PV-EC) system for H_2_S splitting by integrating a single perovskite solar cell, noble-metal-free catalysts and indirect decomposition of H_2_S reaction (As shown in Figure 8a) (Ma et al., 2016). This established system achieved a solar-to-chemical energy conversion efficiency up to 13.5% during the PV-EC step. By employing Fe^2+^/Fe^3+^ as the redox mediator, 0.5 mol/L H_2_SO_4_ as the electrolyte, graphite carbon sheet as the catalyst for oxidation of mediators, and molybdenum-tungsten phosphide (Mo-W-P) material as the cathode electrocatalyst for HER, the total energy consumption for producing an equivalent amount of hydrogen via H_2_S splitting was approximately 43.3% lower than that of conventional water splitting.

To further industrialize the indirect electrolysis of H_2_S technology, Li’s group has implemented modifications to conventional indirect electrolysis processes (Ma D. et al., 2019). They proposed an innovative concept for H_2_S splitting that constructs a solar redox flow battery device based on Fe^2+^/Fe^3+^ and H_4_ [SiW_12_^VI^O_40_]/H_6_ [SiW_10_^VI^W_2_^V^O_40_] redox mediators and perovskite solar cells, which simultaneously stores/utilizes solar energy via charge/discharge redox pairs while decomposing H_2_S into hydrogen and elemental sulfur. As shown in Figure 8b, during the solar-driven charging, energy was stored in redox species by anodic oxidation of Fe^2+^ to Fe^3+^ and the coupled cathodic reduction of H_4_ [SiW_12_^VI^O_40_] to H_6_ [SiW_10_^VI^W_2_^V^O_40_]. Subsequently, the stored chemical energy in the two redox couple pairs was discharged to realize hydrogen and sulfur production. Specifically, the charged Fe^3+^ anolyte was pumped into an absorption reactor to capture H_2_S and convert it into protons and elemental sulfur, whereas the charged H_6_ [SiW_10_^VI^W_2_^V^O_40_] and the protons were pumped into a catalytic hydrogen evolution reactor, which could enable efficient hydrogen production via heterogeneous catalysis with the cobalt phosphide (CoP) catalyst. The constructed flow battery system displayed the stabilized cell voltage at ∼ 0.75 V during the long-term test and achieved a solar-to-chemical energy conversion efficiency of 15.2%. Building on this foundation, Li’s group further developed a solar redox flow battery system based on dual redox couples (Fe^2+^/Fe^3+^ and V^3+^/V^2+^), named as an electron-mediated off-field electrocatalysis approach of H_2_S splitting, which realizes the laboratory scale-up (100 L_H2S_/d) of electrochemical decomposition of H_2_S (Wang Z. et al., 2024). This off-field electrocatalysis system could operatstably over 120 h for a capacity of 100 L_H2S_/d H_2_S, in which H_2_S conversion efficiency is nearly 100%, sulfur-containing pollutant emissions are below 1 ppm, and the energy consumption for hydrogen evolution is estimated to be 2.8 kWh·Nm^-3^ H_2_ (Figure 8c). This technology offers a potential promise for the industrial application of electrocatalytic decomposition of H_2_S.

In addition to the free Fe^2+^/Fe^3+^ redox species in acidic environments, the ethylenediaminetetraacetic acid (EDTA) ligands have been utilized to form iron complexes in alkaline conditions to capture H_2_S and convert it into elemental sulfur, followed by electrochemical regeneration (as illustrated in Figure 8d) (Kim et al., 2013). And the absorption of H_2_S is more favorable, and corrosivity is relatively weaker in alkaline conditions. The required energy input for driving the HER could be reduced due to the lower redox potential of Fe^2+^-EDTA/Fe^3+^-EDTA than that of free Fe^2+^/Fe^3+^ (0.77 V vs. SHE), thereby decreasing the overall energy consumption of H_2_S splitting. Furthermore, the performance of the Fe^2+^-EDTA/Fe^3+^-EDTA redox couple is dramatically influenced by the pH value of the solution. When the pH value is 9, it can efficiently capture H_2_S and maximize the generation of electrical energy (Kim et al., 2013). The reaction equations for the process are as follows (Equations 15, 16):

$$\text{Oxidation of }H_{2}S:2{\left[{\text{Fe}}^{3+}‐\text{EDTA}\right]}^{‐}+{\text{HS}}^{‐}\to \;2{\left[{\text{Fe}}^{2+}‐\text{EDTA}\right]}^{2‐}+H^{+}+S↓$$ (15)

$$\text{Anode}:2{\left[{\text{Fe}}^{2+}‐\text{EDTA}\right]}^{2‐}\;‐\;2e^{‐}\;\to \;2{\left[{\text{Fe}}^{3+}‐\text{EDTA}\right]}^{‐}$$ (16)

#### 3.2.2 I^−^/I_3_^−^ as redox mediators

The I^−^/I_3_^−^ with appropriate redox voltages (0.54 V vs. SHE) used as anodic redox mediator has been proven to facilitate selectively generating sulfur in the solution, effectively mitigating competitive sulfite/sulfate deposition on the electrode interface (Zong et al., 2014). Kalina et al. achieved the indirect electrocatalytic decomposition of H_2_S into elemental sulfur and hydrogen in an acid environment (pH 0–1) by utilizing the I^−^/I_3_^−^ as redox mediators (Kalina, 1985). This redox pair displayed high solubility for both the reduced (I^−^) and oxidized (I_3_^−^) forms with ideal stability, electrochemical activity, and relatively obstructed side reactions. The reaction equations for electrochemical H_2_S splitting using the I^−^/I_3_^−^ redox are presented as follows (Equations 17–19):

$$\text{Oxidation of }H_{2}S:I_{3}^{‐}+H_{2}S\;\to \;2H^{+}+3I^{‐}+S↓$$ (17)

$$\text{Anode}:3I^{‐}\;‐\;2e^{‐}\;\to \;I_{3}^{‐}$$ (18)

$$\text{Cathode}:2H^{+}+2e^{‐}\;\to \;H_{2}↑$$ (19)

**FIGURE 9:**
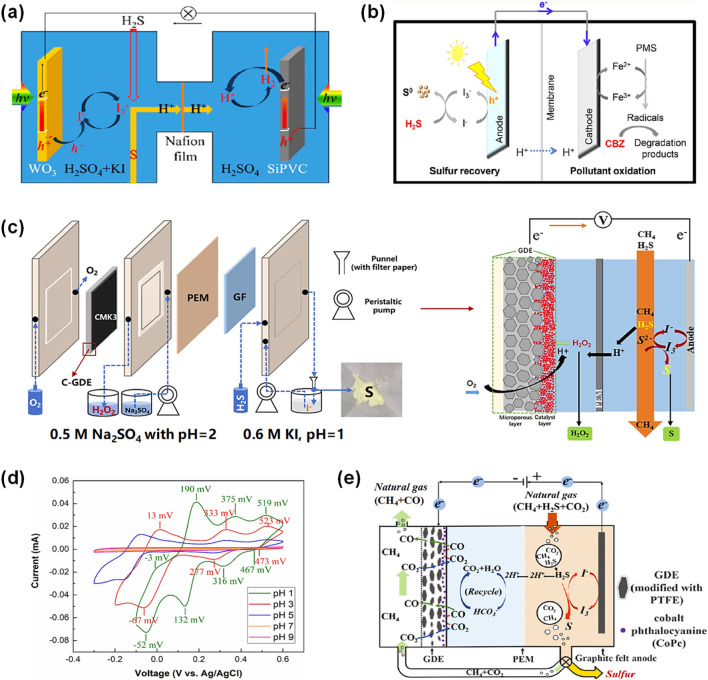
I^−^/I_3_^−^ and polyoxometalates as redox mediators for indirect electrocatalytic decomposition of H_2_S. **(a)** Schematic diagram of a self-driven photoelectrochemical splitting system of H_2_S with I^−^/I_3_^−^ as redox mediator (Luo et al., 2017). **(b)** Schematic diagram of simultaneous recovery of sulfur resources from H_2_S and degradation of organic pollutants by photoelectrocatalysis (Li et al., 2018). **(c)** The designed cell and operation mechanism for indirectly H_2_S conversion to synchronously yield H_2_O_2_ and sulfur (Cui et al., 2024). **(d)** CV curves of 1 mM (NH_4_)_11_ [Sm(PMo_11_O_39_)_2_] on a glassy carbon electrode at various pH values (Li J. et al., 2021). **(e)** Operation principle diagram for the electrocatalytic synergistic conversion of H_2_S and CO_2_ into sulfur and carbon monoxide system (Zhang B. et al., 2021).

During the oxidation of H_2_S, the generated sulfur is in the form of a reddish-brown viscous gel-like precipitate, and the sulfur purity is up to 90% after cooling. The sulfur recovery efficiency can reach 99.3% after treatment with hot toluene. Furthermore, Luo et al. constructed a self-driven photoelectrochemical splitting system of H_2_S into elemental sulfur and hydrogen based on the I^−^/I_3_^−^ as redox mediators, a WO_3_ photoanode and a Si/PVC photocathode (Figure 9a) (Luo et al., 2017). The system exhibited selective oxidation of H_2_S to elemental sulfur without byproduct formation such as polysulfide (S_n_^2-^), attributable to the moderate oxidizing capability of I_3_^−^. Remarkably, high recovery rates of 1.04 mg h^-1^ cm^-2^ for sulfur and 0.75 mL h^-1^ cm^-2^ for hydrogen were achieved, demonstrating near-quantitative conversion of H_2_S to these value-added products.

Moreover, for the indirectly electrocatalytic H_2_S decomposition system, the electrochemical regeneration process with I^−^/I_3_^−^ as redox mediators can not only couple with hydrogen evolution reaction to produce hydrogen, but also pair with Fe^2+^/Fe^3+^ redox couple for organic pollutant degradation. Additionally, it can be integrated with the oxygen reduction reaction to generate hydrogen peroxide (H_2_O_2_). For example, Li et al. proposed a photoelectrochemical approach for the synchronous recovery of sulfur from H_2_S waste gas and removal of organic pollutants (carbamazepine) from wastewater driven by simulated solar light (Li et al., 2018). In this system, H_2_S was selectively converted into high-purity elemental sulfur via the I^−^/I_3_^−^ redox mediator at the photoanode, while carbamazepine pollutant was electrochemically oxidized and degraded at the cathode by Fe^2+^/Fe^3+^-activated peroxymonosulfate (Figure 9b). Notably, the sulfur recovery ratio reached 95.9% and the removal efficiency of carbamazepine pollutant was up to 91.6% within 1 h in this established system. In addition, Cui et al. designed an integrated gas-liquid flow electrocatalytic system to synergistically drive H_2_S oxidation on I^−^/I_3_^−^ redox pairs and O_2_ reduction on the catalyst surface, achieving simultaneous sulfur recovery and H_2_O_2_ production with low energy consumption (Figure 9c) (Cui et al., 2024). By implementing a dual-flow cell configuration integrating a carbon-supported gas diffusion electrode and an I^−^/I_3_^−^ cyclic redox system, a high current density of 102 mA cm^-2^ was achieved, and the corresponding yield rates for elemental sulfur and H_2_O_2_ reached 60 mg cm^-2^ h^-1^ and 50 mg cm^-2^ h^-1^, respectively.

#### 3.2.3 Polyoxometalates as redox mediators

Recently, Polyoxometalates such as PMo_12_O_40_^3−^and (NH_4_)_11_ [Ln (PMo_11_O_39_)_2_] (Ln = Sm, Ce, Dy, or Gd) with appropriate redox potentials and chemical stability, have been used as redox media for efficiently indirect electrocatalytic oxidation of H_2_S into sulfur and hydrogen production with a removing rate of over 90% under optimal conditions (Kim et al., 2014; Li Y. et al., 2021). The reaction equation of H_2_S splitting by employing [Sm(PMo_11_^VI^O_39_)_2_]^−11^/[Sm(PMo_11_^VI^O_39_)_2_]^−55^ redox media as an example is as follows (Equations 20–22) (Kim and Lee, 2023; Li J. et al., 2021):

$$\text{Oxidation of }H_{2}S:{\left[\text{Sm}{\left({\text{PMo}}_{11}^{\text{VI}}O_{39}\right)}_{2}\right]}^{‐11}+22H_{2}S\;\to \;{\left[\text{Sm}{\left({\text{PMo}}_{11}^{\text{VI}}O_{39}\right)}_{2}\right]}^{‐55}+44H^{+}+22S↓$$ (20)

$$\text{Anode}:{\left[\text{Sm}{\left({\text{PMo}}_{11}^{\text{VI}}O_{39}\right)}_{2}\right]}^{‐55}\;‐\;44e^{‐}\;\to \;{\left[\text{Sm}{\left({\text{PMo}}_{11}^{\text{VI}}O_{39}\right)}_{2}\right]}^{‐11}$$ (21)

$$\text{Cathode}:44H^{+}+44e^{‐}\;\to \;22H_{2}↑$$ (22)

Research has demonstrated that the structure and performance of polyoxometalates are significantly influenced by the pH of the solution. The pH-dependent structural variations of polyoxometalates can be visually observed through color changes in the solution from dark yellow to light yellow to colorless. And the UV–vis spectrophotometry and Fourier transform infrared spectroscopy revealed the following transformations of polyoxometalates anions at different pH values (Equation 23) (Da Silva and Teixeira, 2017; Jürgensen and Moffat, 1995; Li J. et al., 2021):

$${\left[{\text{PMo}}_{12}O_{40}\right]}^{3‐}\;\overset{\text{pH}\;>\;1}{\to }\;{\left[{\text{PMo}}_{11}O_{39}\right]}^{7‐}\;\overset{\text{pH}\;>\;7}{\to }\;{\text{MoO}}_{4}^{2‐}+{\text{PO}}_{4}^{3‐}$$ (23)

Furthermore, the influence of pH on the structure and electrochemical performance of polyoxometalates was investigated by cyclic voltammetry (CV). The CV curves shown in Figure 9d indicated the redox peak intensities diminishing with increasing pH. At the lowest tested pH value (pH 1), the polyoxometalates existed in the Keggin structure, and the three largest consecutive redox peaks with each corresponding to a two-electron transfer process were observed, and this process can be illustrated by the following equations (Equation 24) (Lewera et al., 2005):

$${\text{PMo}}_{12}^{\text{VI}}O_{40}^{3‐}+{\text{nH}}^{+}+{\text{ne}}^{‐}\;\to \;H_{n}{\text{PMo}}_{n}^{V}{\text{Mo}}_{12‐n}^{\text{VI}}O_{40}^{3‐}$$ (24)

where n is 2, 4, or 6. Further increasing the pH to 3 and 5, the Keggin structure of PMo_12_^VI^O_40_^3-^ transforms to lacunary-Keggin-type PMo_11_^VI^O_39_^7-^, and three redox peaks with diminished current intensity were shown in the CV curves. These peaks can be explained by the following two-electron transfer processes (Equation 25) (Liu and Dong, 1994):

$${\text{PMo}}_{11}^{\text{VI}}O_{39}^{7‐}+{\text{nH}}^{+}+{\text{ne}}^{‐}\;\to \;H_{n}{\text{PMo}}_{n}^{V}{\text{Mo}}_{11‐n}^{\text{VI}}O_{39}^{7‐}$$ (25)

where n is 2, 4, or 6. When the pH was increased to >7, the redox peaks disappeared due to the decomposition of polyoxometalates. Therefore, the pH-dependent structure and redox activity of polyoxometalates were essential for electrochemically mediated H_2_S capture and anodic regeneration. Additionally, the influence of polyoxometalates concentration on H_2_S removal efficiency was investigated (Kim et al., 2014). At pH 0.8, a lower polyoxometalates concentration (5 mM) resulted in less than 80% H_2_S removal after 1 h, whereas a higher concentration (25 mM) maintained over 90% removal efficiency within 100 min. On the basis of the peak potentials observed in CV curves, it can be inferred that polyoxometalates oxidation at the anode coupled with hydrogen evolution at the cathode in the electrochemical regeneration unit occurred at potentials below 0.8–0.9 V (Kim and Lee, 2023).

#### 3.2.4 Other redox mediators

The (VO_2_)^+^/(VO)^2+^ with an oxidation potential of 0.99 V vs. SHE was also employed as redox media for indirect electrochemical oxidizing H_2_S to elemental sulfur and regenerating at anode (Equations 26–28) (Huang et al., 2009).

$$\text{Oxidation of }H_{2}S:{\left({\text{VO}}_{2}\right)}^{+}+2H^{+}+H_{2}S\;\to \;2{\left(\text{VO}\right)}^{2+}+2H_{2}O+S↓$$ (26)

$$\text{Anode}:2{\left(\text{VO}\right)}^{2+}+2H_{2}O\;‐\;2e^{‐}\;\to \;2{\left({\text{VO}}_{2}\right)}^{+}+4H^{+}$$ (27)

$$\text{Cathode}:2H^{+}+2e^{‐}\;\to \;H_{2}\#$$ (28)

In the acidic vanadium oxo ion solution, the effects of absorption temperature, the molality of H^+^ and (VO)^2+^ in the electrolyte, as well as the molality of (VO_2_)^+^ in the absorption solution on both H_2_S absorption efficiency and electrochemical decomposition efficiency were investigated. The results demonstrate that the H_2_S absorption efficiency increases with rising absorption temperature. When the concentrations of H^+^ and (VO)^2+^ in the electrolyte reached 7.00 mol/kg and 0.65 mol/kg respectively, and the concentration of (VO_2_)^+^ in the absorption process was 0.55 mol/kg, the H_2_S absorption efficiency exceeded 90% at 50 °C, while the current efficiency of the electrochemical regeneration process achieved 97% at 45 °C.

The indirect electrolysis method could decompose H_2_S with different concentration ranges from low to high. By introducing redox mediators, this approach effectively mitigates sulfur passivation on electrodes. Currently, the indirect electrocatalytic splitting of H_2_S process has demonstrated promising results in an expanded test of a certain scale. However, it still faces several challenges, including low mass transfer efficiency for oxidation of H_2_S, corrosion of equipment by acidic electrolytes, mismatched reaction rates between chemical and electrochemical processes, difficulty in separating amorphous sulfur, and short lifespans of electrode materials. Therefore, the key to advancing this technology lies in developing long-lasting, high-performance, and low-cost electrode materials and electrolyzers, designing efficient redox mediators, optimizing process parameters, and improving sulfur recovery efficiency.

## 4 Conclusion and perspective

The electrochemical H_2_S decomposition technology provides dual benefits of waste treatment and energy valorization, serving as an effective approach for achieving clean and high-value utilization of H_2_S resources. In this review, we summarize recent progress in two key research directions: (1) Innovative strategies to mitigate anodic sulfur passivation in direct H_2_S decomposition systems. (2) The development of redox mediators and process optimization for indirect H_2_S decomposition pathway. A comparative analysis of characteristic and reaction mechanism of both approaches is provided, with the aim of providing insights for the development of electrocatalytic H_2_S decomposition technologies.

For the direct electrochemical decomposition of H_2_S, these strategies such as organic solvent treatment, high-temperature melting technique, designing armored electrocatalyst, constructing sulfur-phobic electrocatalytic materials and modulating the anode products have been developed to solve the sulfur passivation at anode. However, the industrial application of this technology still faces several challenges, including the susceptibility of electrode materials to sulfurization at high current densities, poor material stability, and low sulfur recovery efficiency. Future development of this technology may focus on the following aspects: (1) Continuous exploitation of novel electrocatalytic materials with high activity and sulfur-passivation resistance. Transition metal sulfides/selenides are considered optimal choices for SOR catalysts due to their suitable adsorption energy for sulfur-containing species and excellent catalytic activity. High-entropy alloy compounds, with their highly tunable composition and structural advantages, also show great potential in SOR electrocatalysis. Constructing heterojunction catalysts could induce electron redistribution at the interface, generating synergistic effects, which serve as an effective strategy for developing SOR electrocatalysts. Additionally, with the rapid advancement of artificial intelligence in recent years, data-driven approaches such as high-throughput screening and machine learning have provided new research paradigms for the efficient design of SOR electrocatalysts. (2) In-depth understanding of the stability and distribution mechanisms of SOR products. By employing *in situ* characterization techniques to monitor the evolution of reactive intermediates and products (from polysulfides to sulfates) on the catalyst surface in real time, combined with theoretical calculations to elucidate the reaction pathway and mechanism, the sulfur precipitation behavior can be precisely regulated. Furthermore, achieving a single sulfur-containing product through targeted control of the SOR process is crucial for the resource utilization of sulfur, which is a key step toward the industrial application of this technology. (3) Optimization of electrocatalytic devices. High-efficiency electrocatalytic devices play a crucial role in enhancing the H_2_S decomposition performance of the overall reaction system. Membrane electrode assembly (MEA)-based electrolyzers represent an ideal choice for future high-performance electrocatalytic devices, as they can significantly reduce the system impedance while maintaining the mass transport advantages of flow-type electrolyzers. Optimizing the design of electrolyzers and developing high-activity, low-cost and stable membrane electrodes are the development trends for MEA electrolyzers of H_2_S high-efficiency decomposition to meet industrial requirements.

For the indirect electrochemical decomposition of H_2_S, the sulfur passivation at the anode can be effectively mitigated by introducing redox couples as intermediate mediators. Various redox couples such as (VO_2_)^+^/(VO)^2+^, I^−^/I_3_^−^, Fe^2+^/Fe^3+^, and [Sm(PMo^IV^O_39_)_2_]^11-^/[Sm(PMo^V^O_39_)_2_]^55-^ have been developed, which enable efficient H_2_S oxidation and electrochemical coupling for hydrogen production. Furthermore, the conventional indirect process has been optimized, leading to the proposal of an electron-mediated off-field electrocatalysis approach of H_2_S splitting based on dual redox couples (Fe^2+^/Fe^3+^ and V^3+^/V^2+^), and this approach has demonstrated a promising result in a certain scale of expanded experiments. However, the indirect electrolysis of H_2_S still faces several challenges, including low mass transfer efficiency for H_2_S oxidation, corrosion of equipment by acidic electrolytes, difficulty in separating amorphous sulfur, mismatched reaction rates between chemical and electrochemical processes and the short lifespan of electrode materials. Future research directions for this technology may focus on the following aspects: (1) Development of redox mediators with strong oxidizing capacity, high-purity sulfur production with easy separation, and low energy consumption for coupled hydrogen production. (2) Design of high-efficiency absorption reactors to enhance gas-liquid mass transfer and improve H_2_S oxidation rate. (3) Exploitation of highly efficient and stable electrode materials. (4) Process optimization for indirect H_2_S decomposition and development of advanced membrane electrode devices, such as coupling with oxygen reduction reaction to assemble fuel cells, thereby further reducing energy consumption of the process.

Additionally, in the processes of petroleum refining and natural gas purification, substantial carbon dioxide (CO_2_) is concomitantly released alongside H_2_S. The conventional Claus process only purifies the harmful gas H_2_S and recovers sulfur, while neglecting the treatment of the greenhouse gas CO_2_, leading to hydrogen resource wastage and substantial carbon emissions. The electrocatalytic synergistic conversion of H_2_S and CO_2_ provides the potential to produce high-value products (e.g., sulfur, carbonic oxide, methane) under mild reaction conditions, which avoids the generation of byproducts such as carbonyl sulfide and sulfur dioxide, and simultaneously reduces carbon emissions (Fu et al., 2020; Ma et al., 2018; Zhang S. et al., 2021). For example, Zhang et al. constructed a system for the electrocatalytic synergistic conversion of H_2_S and CO_2_ into elemental sulfur and carbon monoxide (Figure 9E), utilizing the I^−^/I_3_^−^ redox mediator and a gas diffusion electrode with polytetrafluoroethylene and cobalt phthalocyanine (CoPc) (Zhang B. et al., 2021). The system achieved production rates of 24.94 mg cm^-2^·h^-1^ for elemental sulfur and 19.93 mL cm^-2^·h^-1^ for carbon monoxide, respectively. Moreover, the model analysis revealed that the operational cost of simultaneous H_2_S and CO_2_ utilization in natural gas purification technology is marginally lower than that of solely utilizing H_2_S. Therefore, the development of H_2_S and CO_2_ co-conversion technology represents a promising future direction for natural gas purification, offering a sustainable pathway for resource utilization and emission reduction.
